# Supplementary material for: Radiosensitization effect of hsa-miR-138-2-3p on human laryngeal cancer stem cells
Source: PeerJ. 2017 May 16;5:e3233. doi: 10.7717/peerj.3233 (PMC5436573; doi:10.7717/peerj.3233)
Supplement: Data S1 [file peerj-05-3233-s001.docx]

**Cell transfection**

**
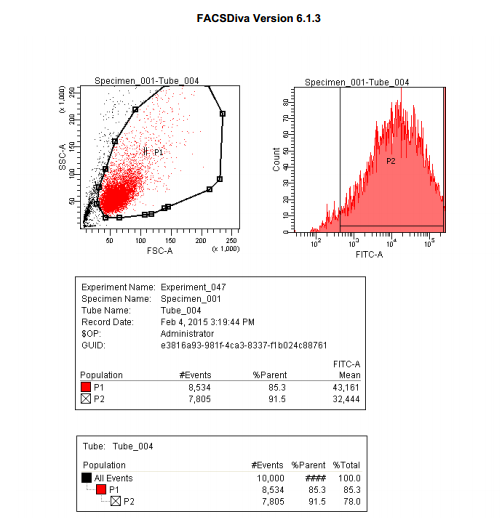

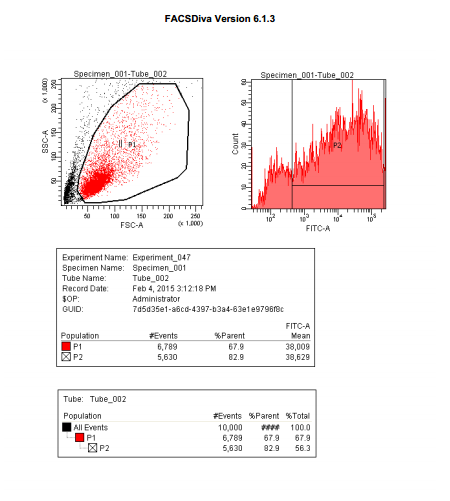
**

**Cell Proliferation**

|  | 50nM-TR | | | 100nM-TR | | | 150nM-TR | | | 100nMN-CR | | | PBS-CR | | |
| --- | --- | --- | --- | --- | --- | --- | --- | --- | --- | --- | --- | --- | --- | --- | --- |
| 0 h | 0.489 | 0.498 | 0.502 | 0.495 | 0.491 | 0.499 | 0.498 | 0.501 | 0.495 | 0.497 | 0.499 | 0.501 | 0.497 | 0.502 | 0.501 |
| 24 h | 0.689 | 0.698 | 0.910 | 0.687 | 0.679 | 0.683 | 0.681 | 0.683 | 0.691 | 0.751 | 0.785 | 0.723 | 0.752 | 0.735 | 0.739 |
| 48 h | 0.812 | 0.814 | 0.811 | 0.798 | 0.795 | 0.789 | 0.779 | 0.789 | 0.788 | 1.223 | 1.175 | 1.189 | 1.216 | 1.211 | 1.207 |
| 72 h | 1.123 | 1.111 | 1.219 | 1.102 | 1.099 | 1.121 | 1.106 | 1.171 | 1.195 | 1.198 | 1.121 | 1.219 | 1.211 | 1.232 | 1.229 |

| Parameter |  |  |  |  |
| --- | --- | --- | --- | --- |
| Table Analyzed | Data 1 |  |  |  |
|  |  |  |  |  |
| Two-way RM ANOVA | Matching by rows |  |  |  |
|  |  |  |  |  |
| Source of Variation | % of total variation | P value |  |  |
| Interaction | 8.89 | < 0.0001 |  |  |
| Row Factor | 84.47 | < 0.0001 |  |  |
| Time | 5.47 | < 0.0001 |  |  |
| Subjects (matching) | 0.2834 | 0.2937 |  |  |
|  |  |  |  |  |
| Source of Variation | P value summary | Significant? |  |  |
| Interaction | *** | Yes |  |  |
| Row Factor | *** | Yes |  |  |
| Time | *** | Yes |  |  |
| Subjects (matching) | ns | No |  |  |
|  |  |  |  |  |
| Source of Variation | Df | Sum-of-squares | Mean square | F |
| Interaction | 12 | 0.3936 | 0.03280 | 26.54 |
| Row Factor | 3 | 3.741 | 1.247 | 794.8 |
| Time | 4 | 0.2422 | 0.06055 | 48.99 |
| Subjects (matching) | 8 | 0.01255 | 0.001569 | 1.269 |
| Residual | 32 | 0.03955 | 0.001236 |  |
|  |  |  |  |  |
| Number of missing values | 0 |  |  |  |
|  |  |  |  |  |
| Bonferroni posttests |  |  |  |  |
|  |  |  |  |  |
| 100nMN-CR vs 50nM-TR |  |  |  |  |
| Row Factor | 100nMN-CR | 50nM-TR | Difference | 95% CI of diff. |
| 0 h | 0.4990 | 0.4963 | -0.002667 | -0.09443 to 0.08909 |
| 24 h | 0.7530 | 0.7657 | 0.01267 | -0.07909 to 0.1044 |
| 48 h | 1.196 | 0.8123 | -0.3833 | -0.4751 to -0.2916 |
| 72 h | 1.179 | 1.151 | -0.02833 | -0.1201 to 0.06343 |
|  |  |  |  |  |
| Row Factor | Difference | t | P value | Summary |
| 0 h | -0.002667 | 0.09289 | P > 0.05 | ns |
| 24 h | 0.01267 | 0.4412 | P > 0.05 | ns |
| 48 h | -0.3833 | 13.35 | P<0.001 | *** |
| 72 h | -0.02833 | 0.9870 | P > 0.05 | ns |
|  |  |  |  |  |
| 100nMN-CR vs 100nM-TR |  |  |  |  |
| Row Factor | 100nMN-CR | 100nM-TR | Difference | 95% CI of diff. |
| 0 h | 0.4990 | 0.4950 | -0.004000 | -0.09576 to 0.08776 |
| 24 h | 0.7530 | 0.6830 | -0.07000 | -0.1618 to 0.02176 |
| 48 h | 1.196 | 0.7940 | -0.4017 | -0.4934 to -0.3099 |
| 72 h | 1.179 | 1.107 | -0.07200 | -0.1638 to 0.01976 |
|  |  |  |  |  |
| Row Factor | Difference | t | P value | Summary |
| 0 h | -0.004000 | 0.1393 | P > 0.05 | ns |
| 24 h | -0.07000 | 2.438 | P > 0.05 | ns |
| 48 h | -0.4017 | 13.99 | P<0.001 | *** |
| 72 h | -0.07200 | 2.508 | P > 0.05 | ns |
|  |  |  |  |  |
| 100nMN-CR vs 150nM-TR |  |  |  |  |
| Row Factor | 100nMN-CR | 150nM-TR | Difference | 95% CI of diff. |
| 0 h | 0.4990 | 0.4980 | -0.001000 | -0.09276 to 0.09076 |
| 24 h | 0.7530 | 0.6850 | -0.06800 | -0.1598 to 0.02376 |
| 48 h | 1.196 | 0.7853 | -0.4103 | -0.5021 to -0.3186 |
| 72 h | 1.179 | 1.157 | -0.02200 | -0.1138 to 0.06976 |
|  |  |  |  |  |
| Row Factor | Difference | t | P value | Summary |
| 0 h | -0.001000 | 0.03483 | P > 0.05 | ns |
| 24 h | -0.06800 | 2.369 | P > 0.05 | ns |
| 48 h | -0.4103 | 14.29 | P<0.001 | *** |
| 72 h | -0.02200 | 0.7664 | P > 0.05 | ns |
|  |  |  |  |  |
| 100nMN-CR vs PBS-CR |  |  |  |  |
| Row Factor | 100nMN-CR | PBS-CR | Difference | 95% CI of diff. |
| 0 h | 0.4990 | 0.5000 | 0.001000 | -0.09076 to 0.09276 |
| 24 h | 0.7530 | 0.7420 | -0.01100 | -0.1028 to 0.08076 |
| 48 h | 1.196 | 1.211 | 0.01567 | -0.07609 to 0.1074 |
| 72 h | 1.179 | 1.224 | 0.04467 | -0.04709 to 0.1364 |
|  |  |  |  |  |
| Row Factor | Difference | t | P value | Summary |
| 0 h | 0.001000 | 0.03484 | P > 0.05 | ns |
| 24 h | -0.01100 | 0.3832 | P > 0.05 | ns |
| 48 h | 0.01567 | 0.5458 | P > 0.05 | ns |
| 72 h | 0.04467 | 1.556 | P > 0.05 | ns |

|  | 100nM-T | | | 100nMN-C | | | PBS-C | | |
| --- | --- | --- | --- | --- | --- | --- | --- | --- | --- |
| 0 h | 0.509 | 0.504 | 0.511 | 0.512 | 0.514 | 0.511 | 0.507 | 0.502 | 0.513 |
| 24 h | 0.801 | 0.798 | 0.804 | 0.814 | 0.807 | 0.812 | 0.816 | 0.809 | 0.821 |
| 48 h | 1.214 | 1.209 | 1.208 | 1.221 | 1.197 | 1.231 | 1.216 | 1.211 | 1.207 |
| 72 h | 1.387 | 1.384 | 1.386 | 1.388 | 1.391 | 1.394 | 1.391 | 1.389 | 1.395 |

| Parameter |  |  |  |  |
| --- | --- | --- | --- | --- |
| Table Analyzed | Data 1 |  |  |  |
|  |  |  |  |  |
| Two-way RM ANOVA | Matching by rows |  |  |  |
|  |  |  |  |  |
| Source of Variation | % of total variation | P value |  |  |
| Interaction | 0.01 | 0.4189 |  |  |
| Row Factor | 99.97 | < 0.0001 |  |  |
| Time | 0.01 | 0.0364 |  |  |
| Subjects (matching) | 0.0089 | 0.2676 |  |  |
|  |  |  |  |  |
| Source of Variation | P value summary | Significant? |  |  |
| Interaction | ns | No |  |  |
| Row Factor | *** | Yes |  |  |
| Time | * | Yes |  |  |
| Subjects (matching) | ns | No |  |  |
|  |  |  |  |  |
| Source of Variation | Df | Sum-of-squares | Mean square | F |
| Interaction | 6 | 0.0002176 | 0.00003627 | 1.072 |
| Row Factor | 3 | 4.254 | 1.418 | 29850 |
| Time | 2 | 0.0002777 | 0.0001389 | 4.104 |
| Subjects (matching) | 8 | 0.0003800 | 0.00004750 | 1.404 |
| Residual | 16 | 0.0005413 | 0.00003383 |  |
|  |  |  |  |  |
| Number of missing values | 0 |  |  |  |
|  |  |  |  |  |
| Bonferroni posttests |  |  |  |  |
|  |  |  |  |  |
| 100nMN-C vs 100nM-T |  |  |  |  |
| Row Factor | 100nMN-C | 100nM-T | Difference | 95% CI of diff. |
| 0 h | 0.5123 | 0.5080 | -0.004333 | -0.01927 to 0.01061 |
| 24 h | 0.8110 | 0.8010 | -0.01000 | -0.02494 to 0.004940 |
| 48 h | 1.216 | 1.210 | -0.006000 | -0.02094 to 0.008940 |
| 72 h | 1.391 | 1.386 | -0.005333 | -0.02027 to 0.009607 |
|  |  |  |  |  |
| Row Factor | Difference | t | P value | Summary |
| 0 h | -0.004333 | 0.9124 | P > 0.05 | ns |
| 24 h | -0.01000 | 2.106 | P > 0.05 | ns |
| 48 h | -0.006000 | 1.263 | P > 0.05 | ns |
| 72 h | -0.005333 | 1.123 | P > 0.05 | ns |
|  |  |  |  |  |
| 100nMN-C vs PBS-C |  |  |  |  |
| Row Factor | 100nMN-C | PBS-C | Difference | 95% CI of diff. |
| 0 h | 0.5123 | 0.5073 | -0.005000 | -0.01994 to 0.009940 |
| 24 h | 0.8110 | 0.8153 | 0.004333 | -0.01061 to 0.01927 |
| 48 h | 1.216 | 1.211 | -0.005000 | -0.01994 to 0.009940 |
| 72 h | 1.391 | 1.392 | 0.0006666 | -0.01427 to 0.01561 |
|  |  |  |  |  |
| Row Factor | Difference | t | P value | Summary |
| 0 h | -0.005000 | 1.053 | P > 0.05 | ns |
| 24 h | 0.004333 | 0.9124 | P > 0.05 | ns |
| 48 h | -0.005000 | 1.053 | P > 0.05 | ns |
| 72 h | 0.0006666 | 0.1404 | P > 0.05 | ns |

**Cell Apoptosis**

**
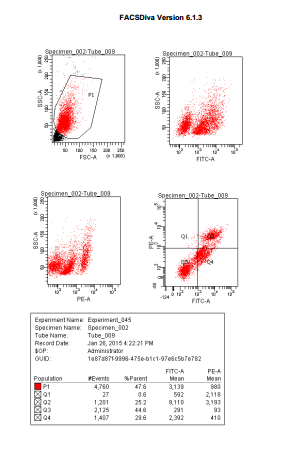

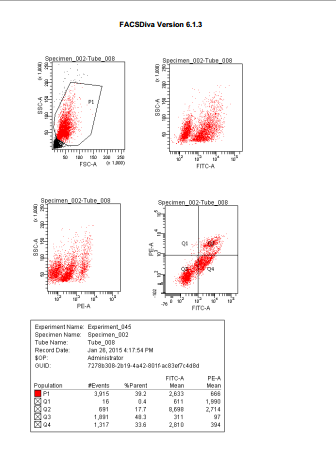

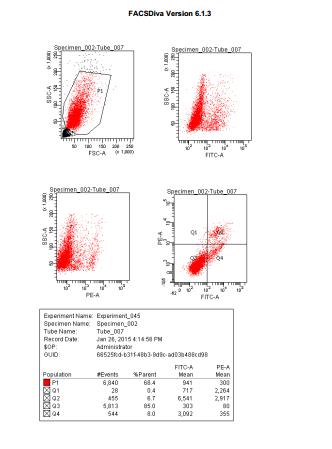

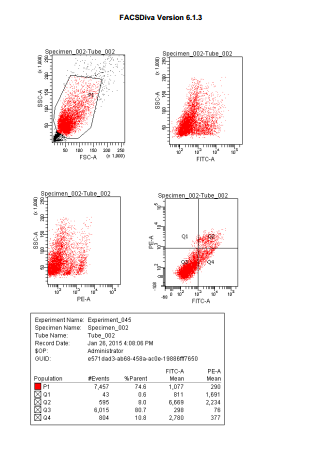
**

**
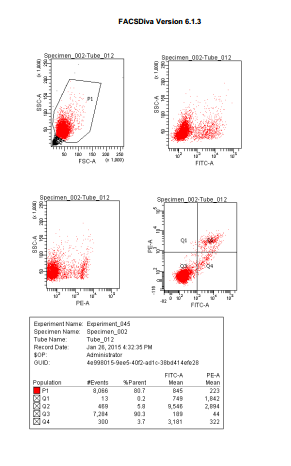

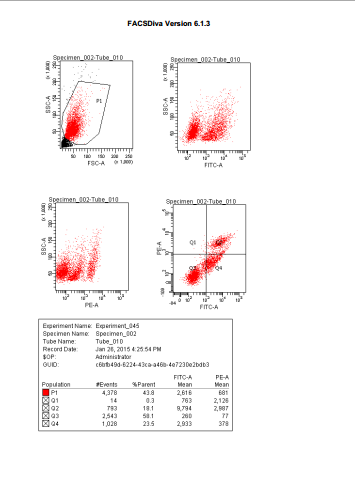
**

|  | HEP-2/100nM-TR | | | HEP-2/100nMN-CR | | | M2E/100nM-TR | | | M2E/100nMN-CR | | | TU212/100nM-TR | | | TU212/100nMN-CR | | |
| --- | --- | --- | --- | --- | --- | --- | --- | --- | --- | --- | --- | --- | --- | --- | --- | --- | --- | --- |
| Early Apoptosis | 1480. | 1432. | 1476. | 540. | 523. | 561. | 1680. | 1654. | 1678. | 400. | 412. | 381. | 1175. | 1156. | 1197. | 185. | 164. | 201. |
| Late Apoptosis | 1260. | 1278. | 1253. | 400. | 423. | 372. | 855. | 825. | 875. | 335. | 321. | 358. | 905. | 915. | 891. | 290. | 271. | 306. |

| Parameter |  |  |  |  |
| --- | --- | --- | --- | --- |
| Table Analyzed | Data 1 |  |  |  |
|  |  |  |  |  |
| Two-way RM ANOVA | Matching by rows |  |  |  |
|  |  |  |  |  |
| Source of Variation | % of total variation | P value |  |  |
| Interaction | 8.87 | < 0.0001 |  |  |
| Row Factor | 5.70 | < 0.0001 |  |  |
| Time | 85.33 | < 0.0001 |  |  |
| Subjects (matching) | 0.0260 | 0.2159 |  |  |
|  |  |  |  |  |
| Source of Variation | P value summary | Significant? |  |  |
| Interaction | *** | Yes |  |  |
| Row Factor | *** | Yes |  |  |
| Time | *** | Yes |  |  |
| Subjects (matching) | ns | No |  |  |
|  |  |  |  |  |
| Source of Variation | Df | Sum-of-squares | Mean square | F |
| Interaction | 5 | 748400 | 149700 | 434.1 |
| Row Factor | 1 | 481200 | 481200 | 878.1 |
| Time | 5 | 7202000 | 1440000 | 4178 |
| Subjects (matching) | 4 | 2192 | 548.0 | 1.589 |
| Residual | 20 | 6896 | 344.8 |  |
|  |  |  |  |  |
| Number of missing values | 0 |  |  |  |
|  |  |  |  |  |
| Bonferroni posttests |  |  |  |  |
|  |  |  |  |  |
| M2E/100nM-TR vs HEP-2/100nM-TR |  |  |  |  |
| Row Factor | M2E/100nM-TR | HEP-2/100nM-TR | Difference | 95% CI of diff. |
| Early Apoptosis | 1671 | 1463 | -208.0 | -255.8 to -160.2 |
| Late Apoptosis | 851.7 | 1264 | 412.0 | 364.2 to 459.8 |
|  |  |  |  |  |
| Row Factor | Difference | t | P value | Summary |
| Early Apoptosis | -208.0 | 13.72 | P<0.001 | *** |
| Late Apoptosis | 412.0 | 27.17 | P<0.001 | *** |
|  |  |  |  |  |
| M2E/100nM-TR vs HEP-2/100nMN-CR |  |  |  |  |
| Row Factor | M2E/100nM-TR | HEP-2/100nMN-CR | Difference | 95% CI of diff. |
| Early Apoptosis | 1671 | 541.3 | -1129 | -1177 to -1082 |
| Late Apoptosis | 851.7 | 398.3 | -453.3 | -501.1 to -405.5 |
|  |  |  |  |  |
| Row Factor | Difference | t | P value | Summary |
| Early Apoptosis | -1129 | 74.49 | P<0.001 | *** |
| Late Apoptosis | -453.3 | 29.90 | P<0.001 | *** |
|  |  |  |  |  |
| M2E/100nM-TR vs M2E/100nMN-CR |  |  |  |  |
| Row Factor | M2E/100nM-TR | M2E/100nMN-CR | Difference | 95% CI of diff. |
| Early Apoptosis | 1671 | 397.7 | -1273 | -1321 to -1225 |
| Late Apoptosis | 851.7 | 338.0 | -513.7 | -561.5 to -465.9 |
|  |  |  |  |  |
| Row Factor | Difference | t | P value | Summary |
| Early Apoptosis | -1273 | 83.96 | P<0.001 | *** |
| Late Apoptosis | -513.7 | 33.88 | P<0.001 | *** |
|  |  |  |  |  |
| M2E/100nM-TR vs TU212/100nM-TR |  |  |  |  |
| Row Factor | M2E/100nM-TR | TU212/100nM-TR | Difference | 95% CI of diff. |
| Early Apoptosis | 1671 | 1176 | -494.7 | -542.5 to -446.9 |
| Late Apoptosis | 851.7 | 903.7 | 52.00 | 4.190 to 99.81 |
|  |  |  |  |  |
| Row Factor | Difference | t | P value | Summary |
| Early Apoptosis | -494.7 | 32.63 | P<0.001 | *** |
| Late Apoptosis | 52.00 | 3.430 | P<0.01 | ** |
|  |  |  |  |  |
| M2E/100nM-TR vs TU212/100nMN-CR |  |  |  |  |
| Row Factor | M2E/100nM-TR | TU212/100nMN-CR | Difference | 95% CI of diff. |
| Early Apoptosis | 1671 | 183.3 | -1487 | -1535 to -1440 |
| Late Apoptosis | 851.7 | 289.0 | -562.7 | -610.5 to -514.9 |
|  |  |  |  |  |
| Row Factor | Difference | t | P value | Summary |
| Early Apoptosis | -1487 | 98.10 | P<0.001 | *** |
| Late Apoptosis | -562.7 | 37.11 | P<0.001 | *** |

| Parameter |  |  |  |  |
| --- | --- | --- | --- | --- |
| Table Analyzed | Data 1 |  |  |  |
|  |  |  |  |  |
| Two-way RM ANOVA | Matching by rows |  |  |  |
|  |  |  |  |  |
| Source of Variation | % of total variation | P value |  |  |
| Interaction | 8.87 | < 0.0001 |  |  |
| Row Factor | 5.70 | < 0.0001 |  |  |
| Time | 85.33 | < 0.0001 |  |  |
| Subjects (matching) | 0.0260 | 0.2159 |  |  |
|  |  |  |  |  |
| Source of Variation | P value summary | Significant? |  |  |
| Interaction | *** | Yes |  |  |
| Row Factor | *** | Yes |  |  |
| Time | *** | Yes |  |  |
| Subjects (matching) | ns | No |  |  |
|  |  |  |  |  |
| Source of Variation | Df | Sum-of-squares | Mean square | F |
| Interaction | 5 | 748400 | 149700 | 434.1 |
| Row Factor | 1 | 481200 | 481200 | 878.1 |
| Time | 5 | 7202000 | 1440000 | 4178 |
| Subjects (matching) | 4 | 2192 | 548.0 | 1.589 |
| Residual | 20 | 6896 | 344.8 |  |
|  |  |  |  |  |
| Number of missing values | 0 |  |  |  |
|  |  |  |  |  |
| Bonferroni posttests |  |  |  |  |
|  |  |  |  |  |
| HEP-2/100nM-TR vs HEP-2/100nMN-CR |  |  |  |  |
| Row Factor | HEP-2/100nM-TR | HEP-2/100nMN-CR | Difference | 95% CI of diff. |
| Early Apoptosis | 1463 | 541.3 | -921.3 | -969.1 to -873.5 |
| Late Apoptosis | 1264 | 398.3 | -865.3 | -913.1 to -817.5 |
|  |  |  |  |  |
| Row Factor | Difference | t | P value | Summary |
| Early Apoptosis | -921.3 | 60.77 | P<0.001 | *** |
| Late Apoptosis | -865.3 | 57.07 | P<0.001 | *** |
|  |  |  |  |  |
| HEP-2/100nM-TR vs M2E/100nM-TR |  |  |  |  |
| Row Factor | HEP-2/100nM-TR | M2E/100nM-TR | Difference | 95% CI of diff. |
| Early Apoptosis | 1463 | 1671 | 208.0 | 160.2 to 255.8 |
| Late Apoptosis | 1264 | 851.7 | -412.0 | -459.8 to -364.2 |
|  |  |  |  |  |
| Row Factor | Difference | t | P value | Summary |
| Early Apoptosis | 208.0 | 13.72 | P<0.001 | *** |
| Late Apoptosis | -412.0 | 27.17 | P<0.001 | *** |
|  |  |  |  |  |
| HEP-2/100nM-TR vs M2E/100nMN-CR |  |  |  |  |
| Row Factor | HEP-2/100nM-TR | M2E/100nMN-CR | Difference | 95% CI of diff. |
| Early Apoptosis | 1463 | 397.7 | -1065 | -1113 to -1017 |
| Late Apoptosis | 1264 | 338.0 | -925.7 | -973.5 to -877.9 |
|  |  |  |  |  |
| Row Factor | Difference | t | P value | Summary |
| Early Apoptosis | -1065 | 70.24 | P<0.001 | *** |
| Late Apoptosis | -925.7 | 61.05 | P<0.001 | *** |
|  |  |  |  |  |
| HEP-2/100nM-TR vs TU212/100nM-TR |  |  |  |  |
| Row Factor | HEP-2/100nM-TR | TU212/100nM-TR | Difference | 95% CI of diff. |
| Early Apoptosis | 1463 | 1176 | -286.7 | -334.5 to -238.9 |
| Late Apoptosis | 1264 | 903.7 | -360.0 | -407.8 to -312.2 |
|  |  |  |  |  |
| Row Factor | Difference | t | P value | Summary |
| Early Apoptosis | -286.7 | 18.91 | P<0.001 | *** |
| Late Apoptosis | -360.0 | 23.74 | P<0.001 | *** |
|  |  |  |  |  |
| HEP-2/100nM-TR vs TU212/100nMN-CR |  |  |  |  |
| Row Factor | HEP-2/100nM-TR | TU212/100nMN-CR | Difference | 95% CI of diff. |
| Early Apoptosis | 1463 | 183.3 | -1279 | -1327 to -1232 |
| Late Apoptosis | 1264 | 289.0 | -974.7 | -1022 to -926.9 |
|  |  |  |  |  |
| Row Factor | Difference | t | P value | Summary |
| Early Apoptosis | -1279 | 84.38 | P<0.001 | *** |
| Late Apoptosis | -974.7 | 64.29 | P<0.001 | *** |

| Parameter |  |  |  |  |
| --- | --- | --- | --- | --- |
| Table Analyzed | Data 1 |  |  |  |
|  |  |  |  |  |
| Two-way RM ANOVA | Matching by rows |  |  |  |
|  |  |  |  |  |
| Source of Variation | % of total variation | P value |  |  |
| Interaction | 8.87 | < 0.0001 |  |  |
| Row Factor | 5.70 | < 0.0001 |  |  |
| Time | 85.33 | < 0.0001 |  |  |
| Subjects (matching) | 0.0260 | 0.2159 |  |  |
|  |  |  |  |  |
| Source of Variation | P value summary | Significant? |  |  |
| Interaction | *** | Yes |  |  |
| Row Factor | *** | Yes |  |  |
| Time | *** | Yes |  |  |
| Subjects (matching) | ns | No |  |  |
|  |  |  |  |  |
| Source of Variation | Df | Sum-of-squares | Mean square | F |
| Interaction | 5 | 748400 | 149700 | 434.1 |
| Row Factor | 1 | 481200 | 481200 | 878.1 |
| Time | 5 | 7202000 | 1440000 | 4178 |
| Subjects (matching) | 4 | 2192 | 548.0 | 1.589 |
| Residual | 20 | 6896 | 344.8 |  |
|  |  |  |  |  |
| Number of missing values | 0 |  |  |  |
|  |  |  |  |  |
| Bonferroni posttests |  |  |  |  |
|  |  |  |  |  |
| TU212/100nM-TR vs HEP-2/100nM-TR |  |  |  |  |
| Row Factor | TU212/100nM-TR | HEP-2/100nM-TR | Difference | 95% CI of diff. |
| Early Apoptosis | 1176 | 1463 | 286.7 | 238.9 to 334.5 |
| Late Apoptosis | 903.7 | 1264 | 360.0 | 312.2 to 407.8 |
|  |  |  |  |  |
| Row Factor | Difference | t | P value | Summary |
| Early Apoptosis | 286.7 | 18.91 | P<0.001 | *** |
| Late Apoptosis | 360.0 | 23.74 | P<0.001 | *** |
|  |  |  |  |  |
| TU212/100nM-TR vs HEP-2/100nMN-CR |  |  |  |  |
| Row Factor | TU212/100nM-TR | HEP-2/100nMN-CR | Difference | 95% CI of diff. |
| Early Apoptosis | 1176 | 541.3 | -634.7 | -682.5 to -586.9 |
| Late Apoptosis | 903.7 | 398.3 | -505.3 | -553.1 to -457.5 |
|  |  |  |  |  |
| Row Factor | Difference | t | P value | Summary |
| Early Apoptosis | -634.7 | 41.86 | P<0.001 | *** |
| Late Apoptosis | -505.3 | 33.33 | P<0.001 | *** |
|  |  |  |  |  |
| TU212/100nM-TR vs M2E/100nM-TR |  |  |  |  |
| Row Factor | TU212/100nM-TR | M2E/100nM-TR | Difference | 95% CI of diff. |
| Early Apoptosis | 1176 | 1671 | 494.7 | 446.9 to 542.5 |
| Late Apoptosis | 903.7 | 851.7 | -52.00 | -99.81 to -4.190 |
|  |  |  |  |  |
| Row Factor | Difference | t | P value | Summary |
| Early Apoptosis | 494.7 | 32.63 | P<0.001 | *** |
| Late Apoptosis | -52.00 | 3.430 | P<0.01 | ** |
|  |  |  |  |  |
| TU212/100nM-TR vs M2E/100nMN-CR |  |  |  |  |
| Row Factor | TU212/100nM-TR | M2E/100nMN-CR | Difference | 95% CI of diff. |
| Early Apoptosis | 1176 | 397.7 | -778.3 | -826.1 to -730.5 |
| Late Apoptosis | 903.7 | 338.0 | -565.7 | -613.5 to -517.9 |
|  |  |  |  |  |
| Row Factor | Difference | t | P value | Summary |
| Early Apoptosis | -778.3 | 51.34 | P<0.001 | *** |
| Late Apoptosis | -565.7 | 37.31 | P<0.001 | *** |
|  |  |  |  |  |
| TU212/100nM-TR vs TU212/100nMN-CR |  |  |  |  |
| Row Factor | TU212/100nM-TR | TU212/100nMN-CR | Difference | 95% CI of diff. |
| Early Apoptosis | 1176 | 183.3 | -992.7 | -1040 to -944.9 |
| Late Apoptosis | 903.7 | 289.0 | -614.7 | -662.5 to -566.9 |
|  |  |  |  |  |
| Row Factor | Difference | t | P value | Summary |
| Early Apoptosis | -992.7 | 65.47 | P<0.001 | *** |
| Late Apoptosis | -614.7 | 40.54 | P<0.001 | *** |

**Cell Cycle**

**
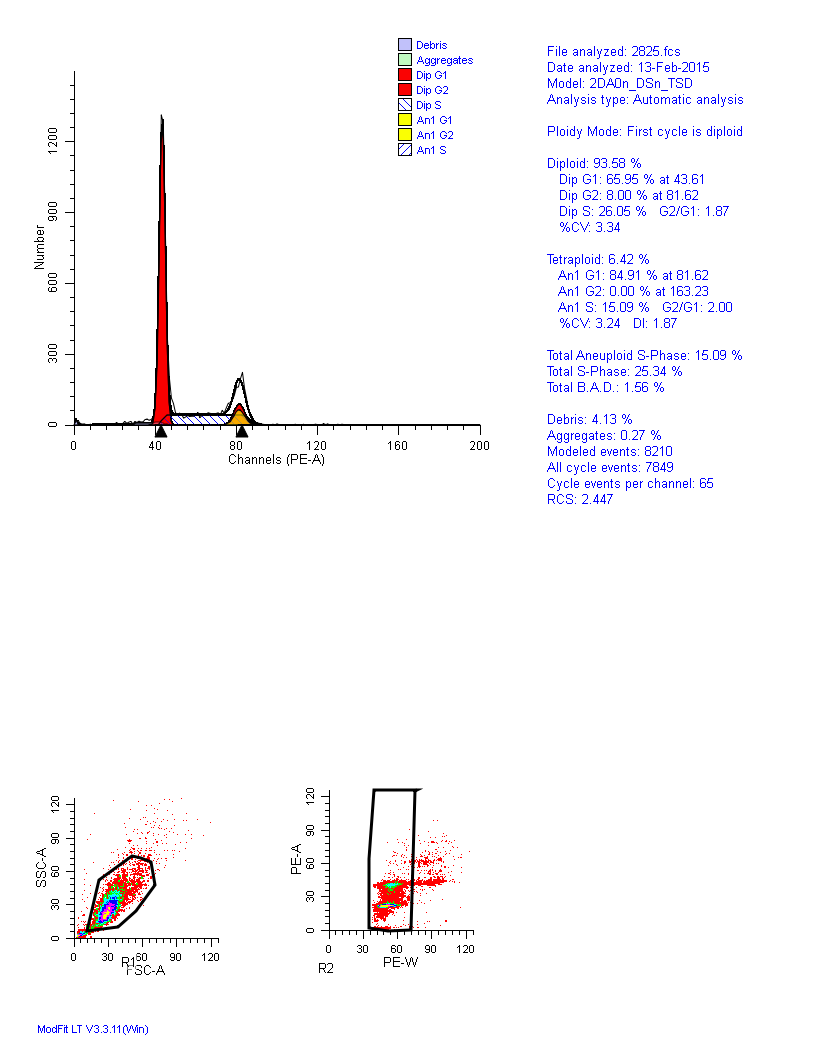

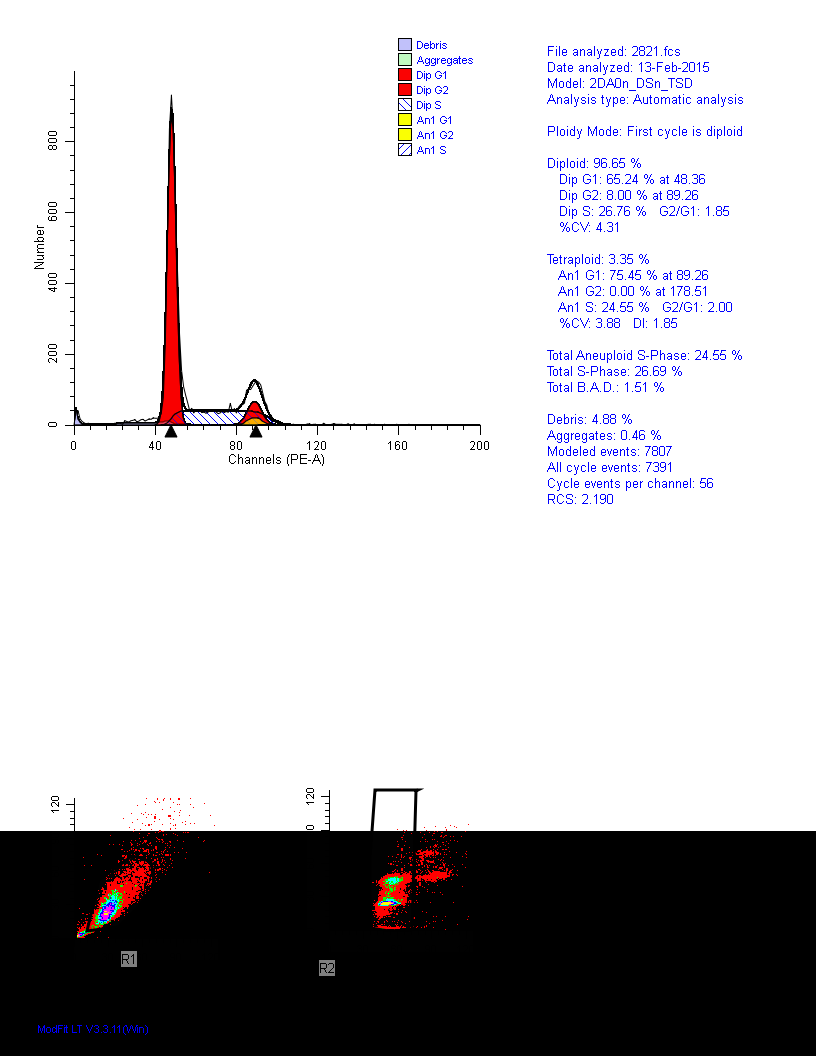

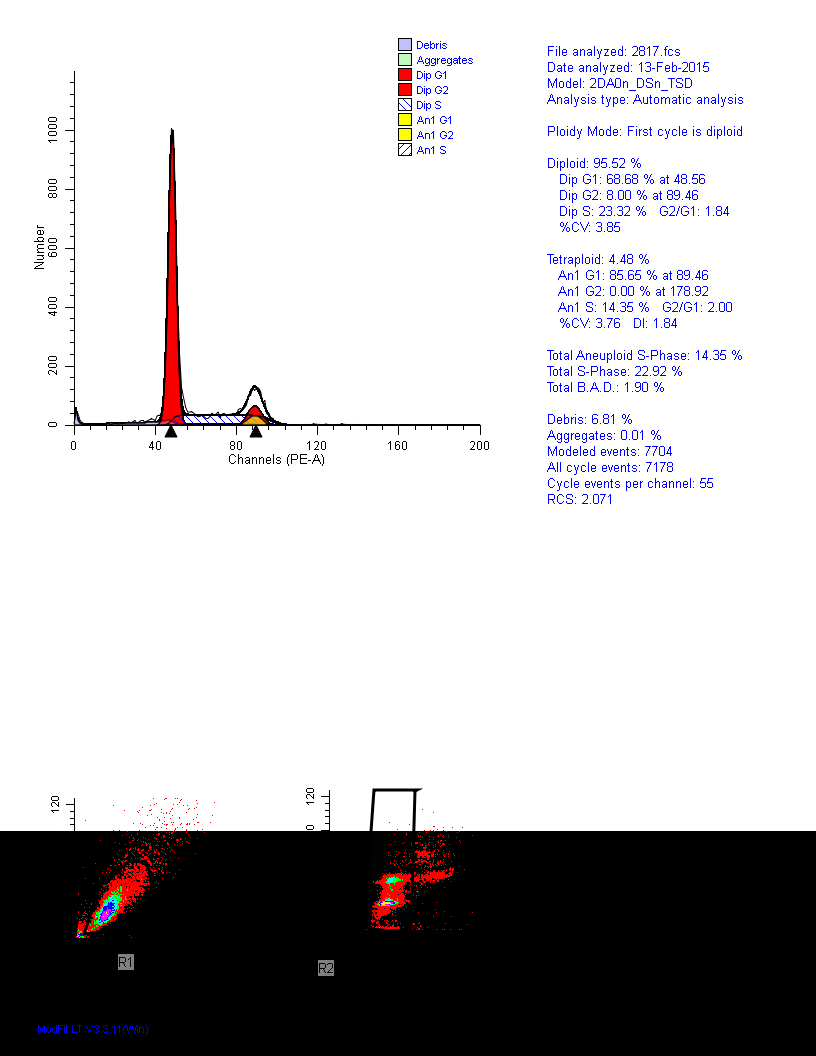
**

**
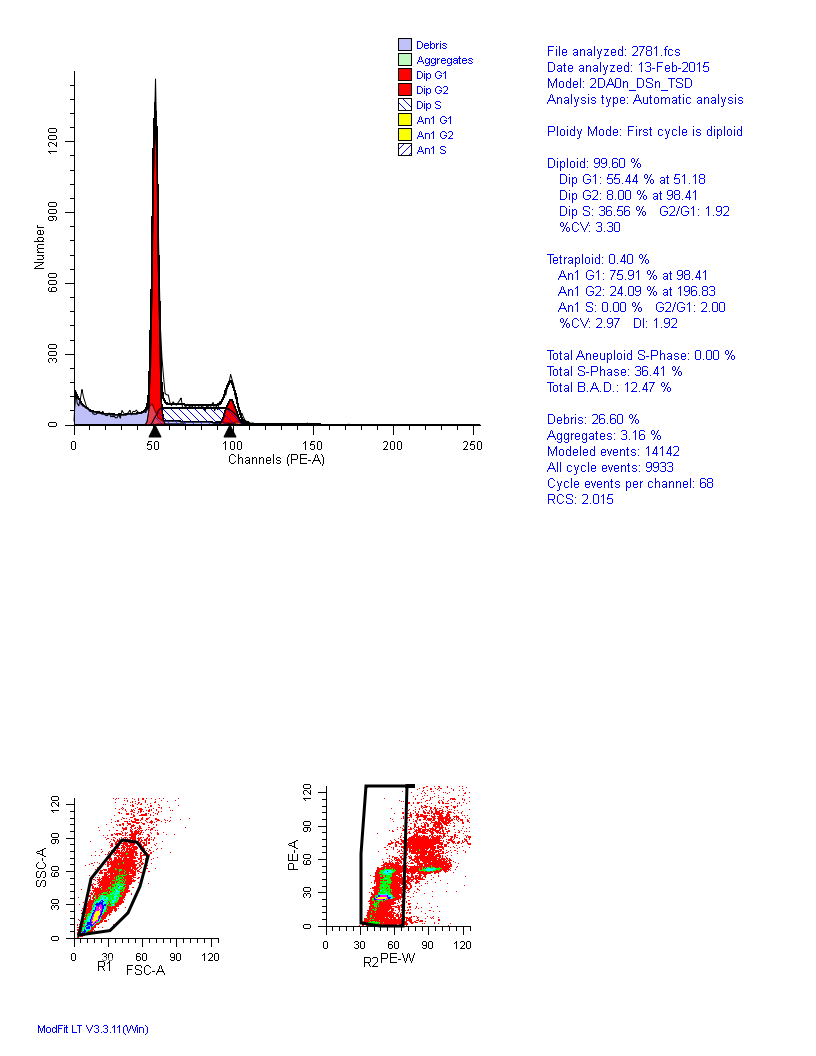

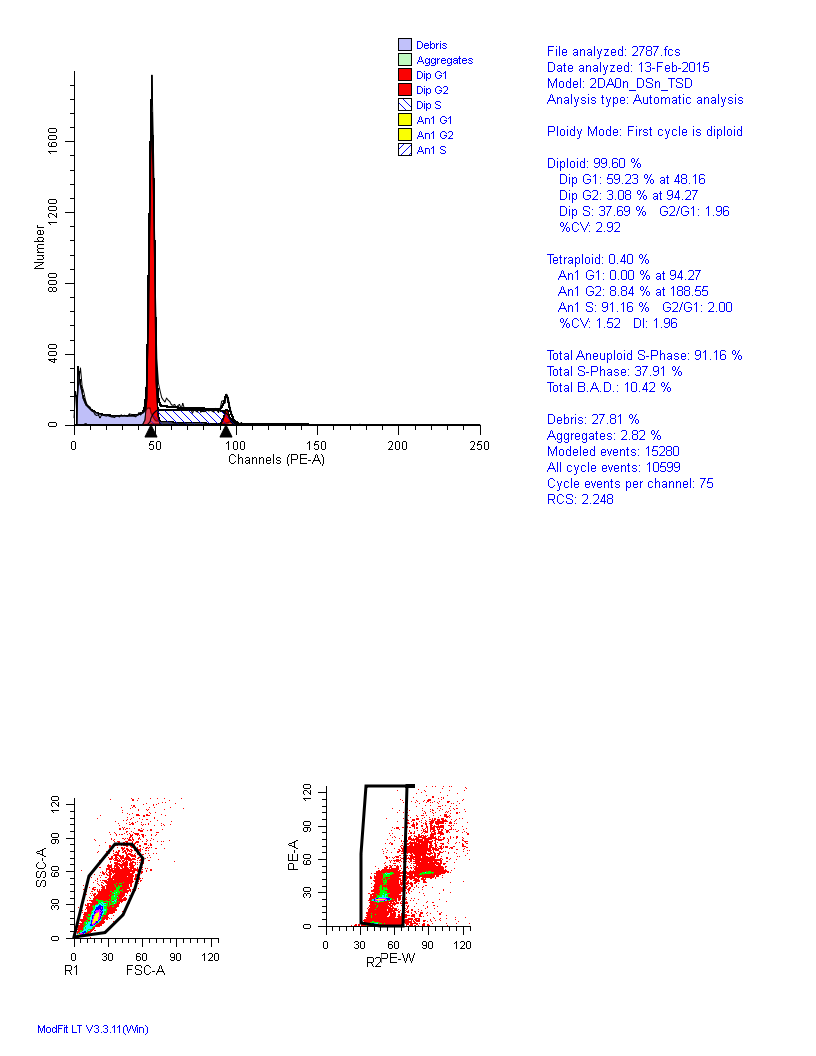

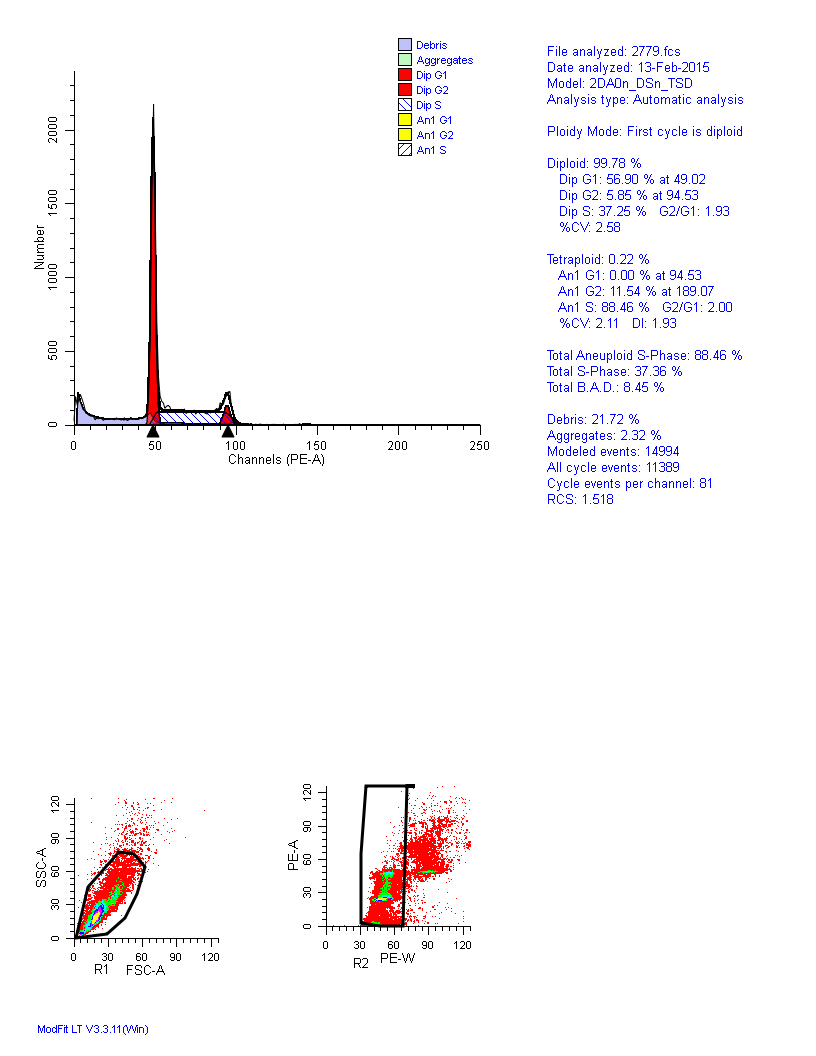
**

|  | HEP-2/100nM-TR | HEP-2/100nMN-CR | M2E/100nM-TR | M2E/100nMN-CR | TU212/100nM-TR | TU212/100nMN-CR |
| --- | --- | --- | --- | --- | --- | --- |
| G1 | 69 | 55 | 66 | 57 | 65 | 59 |
| S | 23 | 37 | 26 | 37 | 27 | 38 |
| G2/M | 8 | 8 | 8 | 6 | 8 | 3 |

**Transwell Assay**

| 100nMN-T | | | 100nMN-C | | | | | | PBS-C | | | | |  |  |  |  |  |  |  |
| --- | --- | --- | --- | --- | --- | --- | --- | --- | --- | --- | --- | --- | --- | --- | --- | --- | --- | --- | --- | --- |
| 212. | 201. | 229. | 226. | | 207. | | 239. | | 228. | 211. | | 242. | |  |  |  |  |  |  |  |
|  |  |  |  | |  | |  | |  |  | |  | |  |  |  |  |  |  |  |
| Parameter | | | |  | |  | |  | | |  | |  | |  |  |  |  |  |  |
| Table Analyzed | | | | Data 1 | |  | |  | | |  | |  | |  |  |  |  |  |  |
|  | | | |  | |  | |  | | |  | |  | |  |  |  |  |  |  |
| One-way analysis of variance | | | |  | |  | |  | | |  | |  | |  |  |  |  |  |  |
| P value | | | | 0.5804 | |  | |  | | |  | |  | |  |  |  |  |  |  |
| P value summary | | | | ns | |  | |  | | |  | |  | |  |  |  |  |  |  |
| Are means signif. different? (P < 0.05) | | | | No | |  | |  | | |  | |  | |  |  |  |  |  |  |
| Number of groups | | | | 3 | |  | |  | | |  | |  | |  |  |  |  |  |  |
| F | | | | 0.5966 | |  | |  | | |  | |  | |  |  |  |  |  |  |
| R squared | | | | 0.1659 | |  | |  | | |  | |  | |  |  |  |  |  |  |
|  | | | |  | |  | |  | | |  | |  | |  |  |  |  |  |  |
| ANOVA Table | | | | SS | | df | | MS | | |  | |  | |  |  |  |  |  |  |
| Treatment (between columns) | | | | 278.0 | | 2 | | 139.0 | | |  | |  | |  |  |  |  |  |  |
| Residual (within columns) | | | | 1398 | | 6 | | 233.0 | | |  | |  | |  |  |  |  |  |  |
| Total | | | | 1676 | | 8 | |  | | |  | |  | |  |  |  |  |  |  |
|  | | | |  | |  | |  | | |  | |  | |  |  |  |  |  |  |
| Tukey's Multiple Comparison Test | | | | Mean Diff. | | q | | Significant? P < 0.05? | | | Summary | | 95% CI of diff | |  |  |  |  |  |  |
| 100nMN-T vs 100nMN-C | | | | -10.00 | | 1.135 | | No | | | ns | | -48.24 to 28.24 | |  |  |  |  |  |  |
| 100nMN-T vs PBS-C | | | | -13.00 | | 1.475 | | No | | | ns | | -51.24 to 25.24 | |  |  |  |  |  |  |
| 100nMN-C vs PBS-C | | | | -3.000 | | 0.3404 | | No | | | ns | | -41.24 to 35.24 | |  |  |  |  |  |  |
|  | | | |  | |  | |  | | |  | |  | |  |  |  |  |  |  |
| 50nM-TR | | | 100nM-TR | | | | | | 150nM-TR | | | | | 100nMN-CR | | | | PBS-CR | | |
| 121. | 132. | 116. | 41. | | 37. | | 42. | | 40. | 39. | | 42. | | 168. | | 154. | 172. | 187. | 175. | 193. |

| Parameter |  |  |  |  |  |
| --- | --- | --- | --- | --- | --- |
| Table Analyzed | Data 1 |  |  |  |  |
|  |  |  |  |  |  |
| One-way analysis of variance |  |  |  |  |  |
| P value | < 0.0001 |  |  |  |  |
| P value summary | *** |  |  |  |  |
| Are means signif. different? (P < 0.05) | Yes |  |  |  |  |
| Number of groups | 5 |  |  |  |  |
| F | 278.4 |  |  |  |  |
| R squared | 0.9911 |  |  |  |  |
|  |  |  |  |  |  |
| ANOVA Table | SS | df | MS |  |  |
| Treatment (between columns) | 55600 | 4 | 13900 |  |  |
| Residual (within columns) | 499.3 | 10 | 49.93 |  |  |
| Total | 56100 | 14 |  |  |  |
|  |  |  |  |  |  |
| Tukey's Multiple Comparison Test | Mean Diff. | q | Significant? P < 0.05? | Summary | 95% CI of diff |
| 50nM-TR vs 100nM-TR | 83.00 | 20.34 | Yes | *** | 64.01 to 102.0 |
| 50nM-TR vs 150nM-TR | 82.67 | 20.26 | Yes | *** | 63.68 to 101.7 |
| 50nM-TR vs 100nMN-CR | -41.67 | 10.21 | Yes | *** | -60.65 to -22.68 |
| 50nM-TR vs PBS-CR | -62.00 | 15.20 | Yes | *** | -80.99 to -43.01 |
| 100nM-TR vs 150nM-TR | -0.3333 | 0.08170 | No | ns | -19.32 to 18.65 |
| 100nM-TR vs 100nMN-CR | -124.7 | 30.56 | Yes | *** | -143.7 to -105.7 |
| 100nM-TR vs PBS-CR | -145.0 | 35.54 | Yes | *** | -164.0 to -126.0 |
| 150nM-TR vs 100nMN-CR | -124.3 | 30.48 | Yes | *** | -143.3 to -105.3 |
| 150nM-TR vs PBS-CR | -144.7 | 35.46 | Yes | *** | -163.7 to -125.7 |
| 100nMN-CR vs PBS-CR | -20.33 | 4.984 | Yes | * | -39.32 to -1.346 |

**Survival Fraction**

| \| Gy \| HEP-2 non-transfection \| 100nMN-CR \| 100nM-TR \| \| --- \| --- \| --- \| --- \| \| 0. \| 1 \| 1 \| 1 \| \| 0. \| 1 \| 1 \| 1 \| \| 0. \| 1 \| 1 \| 1 \| \| 0. \| 1 \| 1 \| 1 \| \| 0. \| 1 \| 1 \| 1 \| \| 0. \| 1 \| 1 \| 1 \| \| 0. \| 1 \| 1 \| 1 \| \| 0. \| 1 \| 1 \| 1 \| \| 0. \| 0 \| 0 \| 0 \| \| 0. \| 1 \| 0 \| 0 \| \| 0. \| 1 \| 0 \| 0 \| \| 0. \| 1 \| 0 \| 0 \| \| 0. \| 1 \| 1 \| 0 \| \| 2. \| 1 \| 1 \| 1 \| \| 2. \| 1 \| 1 \| 1 \| \| 2. \| 1 \| 1 \| 1 \| \| 2. \| 1 \| 1 \| 1 \| \| 2. \| 1 \| 1 \| 1 \| \| 2. \| 1 \| 1 \| 0 \| \| 2. \| 1 \| 0 \| 0 \| \| 2. \| 0 \| 0 \| 0 \| \| 2. \| 0 \| 0 \| 0 \| \| 2. \| 0 \| 0 \| 0 \| \| 2. \| 1 \| 1 \| 0 \| \| 2. \| 1 \| 0 \| 0 \| \| 2. \| 1 \| 0 \| 0 \| \| 2. \| 1 \| 1 \| 0 \| \| 4. \| 1 \| 1 \| 1 \| \| 4. \| 1 \| 1 \| 1 \| \| 4. \| 1 \| 1 \| 1 \| \| 4. \| 1 \| 1 \| 0 \| \| 4. \| 1 \| 0 \| 0 \| \| 4. \| 0 \| 0 \| 0 \| \| 4. \| 0 \| 0 \| 0 \| \| 4. \| 0 \| 0 \| 0 \| \| 4. \| 0 \| 0 \| 0 \| \| 4. \| 0 \| 0 \| 0 \| \| 4. \| 1 \| 0 \| 0 \| \| 4. \| 1 \| 1 \| 0 \| \| 4. \| 1 \| 0 \| 0 \| \| 4. \| 1 \| 1 \| 0 \| \| 6. \| 1 \| 1 \| 1 \| \| 6. \| 1 \| 1 \| 1 \| \| 6. \| 1 \| 0 \| 0 \| \| 6. \| 1 \| 0 \| 0 \| \| 6. \| 0 \| 0 \| 0 \| \| 6. \| 0 \| 0 \| 0 \| \| 6. \| 0 \| 0 \| 0 \| \| 6. \| 0 \| 0 \| 0 \| \| 6. \| 0 \| 0 \| 0 \| \| 6. \| 0 \| 0 \| 0 \| \| 6. \| 1 \| 0 \| 0 \| \| 6. \| 1 \| 1 \| 0 \| \| 6. \| 1 \| 0 \| 0 \| \| 6. \| 1 \| 1 \| 0 \| \| 8. \| 1 \| 1 \| 0 \| \| 8. \| 1 \| 0 \| 0 \| \| 8. \| 1 \| 0 \| 0 \| \| 8. \| 0 \| 0 \| 0 \| \| 8. \| 0 \| 0 \| 0 \| \| 8. \| 0 \| 0 \| 0 \| \| 8. \| 0 \| 0 \| 0 \| \| 8. \| 0 \| 0 \| 0 \| \| 8. \| 0 \| 0 \| 0 \| \| 8. \| 0 \| 0 \| 0 \| \| 8. \| 1 \| 0 \| 0 \| \| 8. \| 1 \| 1 \| 0 \| \| 8. \| 1 \| 0 \| 0 \| \| 8. \| 1 \| 1 \| 0 \| |  |  |  |
| --- | --- | --- | --- | --- | --- | --- | --- | --- | --- | --- | --- | --- | --- | --- | --- | --- | --- | --- | --- | --- | --- | --- | --- | --- | --- | --- | --- | --- | --- | --- | --- | --- | --- | --- | --- | --- | --- | --- | --- | --- | --- | --- | --- | --- | --- | --- | --- | --- | --- | --- | --- | --- | --- | --- | --- | --- | --- | --- | --- | --- | --- | --- | --- | --- | --- | --- | --- | --- | --- | --- | --- | --- | --- | --- | --- | --- | --- | --- | --- | --- | --- | --- | --- | --- | --- | --- | --- | --- | --- | --- | --- | --- | --- | --- | --- | --- | --- | --- | --- | --- | --- | --- | --- | --- | --- | --- | --- | --- | --- | --- | --- | --- | --- | --- | --- | --- | --- | --- | --- | --- | --- | --- | --- | --- | --- | --- | --- | --- | --- | --- | --- | --- | --- | --- | --- | --- | --- | --- | --- | --- | --- | --- | --- | --- | --- | --- | --- | --- | --- | --- | --- | --- | --- | --- | --- | --- | --- | --- | --- | --- | --- | --- | --- | --- | --- | --- | --- | --- | --- | --- | --- | --- | --- | --- | --- | --- | --- | --- | --- | --- | --- | --- | --- | --- | --- | --- | --- | --- | --- | --- | --- | --- | --- | --- | --- | --- | --- | --- | --- | --- | --- | --- | --- | --- | --- | --- | --- | --- | --- | --- | --- | --- | --- | --- | --- | --- | --- | --- | --- | --- | --- | --- | --- | --- | --- | --- | --- | --- | --- | --- | --- | --- | --- | --- | --- | --- | --- | --- | --- | --- | --- | --- | --- | --- | --- | --- | --- | --- | --- | --- | --- | --- | --- | --- | --- | --- | --- | --- | --- | --- | --- | --- | --- | --- | --- | --- | --- | --- | --- | --- | --- | --- | --- | --- | --- | --- | --- | --- | --- | --- | --- | --- | --- |
|  |  |  |  |
|  |  |  |  |
|  |  |  |  |
|  |  |  |  |
|  |  |  |  |
|  |  |  |  |
|  |  |  |  |
|  |  |  |  |
|  |  |  |  |
|  |  |  |  |
|  |  |  |  |
|  |  |  |  |
|  |  |  |  |
|  |  |  |  |
|  |  |  |  |
|  |  |  |  |
|  |  |  |  |
|  |  |  |  |
|  |  |  |  |
|  |  |  |  |
|  |  |  |  |
|  |  |  |  |
|  |  |  |  |
|  |  |  |  |
|  |  |  |  |
|  |  |  |  |
|  |  |  |  |
|  |  |  |  |
|  |  |  |  |
|  |  |  |  |
|  |  |  |  |
|  |  |  |  |
|  |  |  |  |
|  |  |  |  |
|  |  |  |  |
|  |  |  |  |
|  |  |  |  |
|  |  |  |  |
|  |  |  |  |
|  |  |  |  |
|  |  |  |  |
|  |  |  |  |
|  |  |  |  |
|  |  |  |  |
|  |  |  |  |
|  |  |  |  |
|  |  |  |  |
|  |  |  |  |
|  |  |  |  |
|  |  |  |  |
|  |  |  |  |
|  |  |  |  |
|  |  |  |  |
|  |  |  |  |
|  |  |  |  |
|  |  |  |  |
|  |  |  |  |
|  |  |  |  |
|  |  |  |  |
|  |  |  |  |
|  |  |  |  |
|  |  |  |  |
|  |  |  |  |
|  |  |  |  |
|  |  |  |  |
|  |  |  |  |
|  |  |  |  |
|  |  |  |  |
|  |  |  |  |
| Gy | M2E non-transfection | 100nMN-CR | 100nM-TR |
| 0. | 1 | 1 | 1 |
| 0. | 1 | 1 | 1 |
| 0. | 1 | 1 | 1 |
| 0. | 1 | 1 | 1 |
| 0. | 1 | 1 | 1 |
| 0. | 1 | 1 | 1 |
| 0. | 1 | 1 | 1 |
| 0. | 1 | 1 | 1 |
| 0. | 0 | 0 | 0 |
| 0. | 1 | 0 | 0 |
| 0. | 1 | 0 | 0 |
| 0. | 1 | 0 | 0 |
| 0. | 1 | 1 | 0 |
| 0. | 1 | 1 | 0 |
| 0. | 1 | 0 | 0 |
| 2. | 1 | 1 | 1 |
| 2. | 1 | 1 | 1 |
| 2. | 1 | 1 | 1 |
| 2. | 1 | 1 | 1 |
| 2. | 1 | 1 | 1 |
| 2. | 1 | 1 | 0 |
| 2. | 1 | 0 | 0 |
| 2. | 0 | 0 | 0 |
| 2. | 0 | 0 | 0 |
| 2. | 0 | 0 | 0 |
| 2. | 1 | 1 | 0 |
| 2. | 1 | 0 | 0 |
| 2. | 1 | 0 | 0 |
| 2. | 1 | 1 | 0 |
| 2. | 1 | 0 | 0 |
| 2. | 1 | 1 | 0 |
| 4. | 1 | 1 | 1 |
| 4. | 1 | 1 | 1 |
| 4. | 1 | 1 | 1 |
| 4. | 1 | 1 | 0 |
| 4. | 1 | 0 | 0 |
| 4. | 0 | 0 | 0 |
| 4. | 0 | 0 | 0 |
| 4. | 0 | 0 | 0 |
| 4. | 0 | 0 | 0 |
| 4. | 0 | 0 | 0 |
| 4. | 1 | 0 | 0 |
| 4. | 1 | 1 | 0 |
| 4. | 1 | 0 | 0 |
| 4. | 1 | 1 | 0 |
| 4. | 1 | 0 | 0 |
| 4. | 1 | 1 | 0 |
| 6. | 1 | 1 | 1 |
| 6. | 1 | 1 | 1 |
| 6. | 1 | 0 | 0 |
| 6. | 1 | 0 | 0 |
| 6. | 0 | 0 | 0 |
| 6. | 0 | 0 | 0 |
| 6. | 0 | 0 | 0 |
| 6. | 0 | 0 | 0 |
| 6. | 0 | 0 | 0 |
| 6. | 0 | 0 | 0 |
| 6. | 1 | 0 | 0 |
| 6. | 1 | 1 | 0 |
| 6. | 1 | 0 | 0 |
| 6. | 1 | 1 | 0 |
| 6. | 1 | 0 | 0 |
| 6. | 1 | 1 | 0 |
| 8. | 1 | 1 | 0 |
| 8. | 1 | 0 | 0 |
| 8. | 1 | 0 | 0 |
| 8. | 0 | 0 | 0 |
| 8. | 0 | 0 | 0 |
| 8. | 0 | 0 | 0 |
| 8. | 0 | 0 | 0 |
| 8. | 0 | 0 | 0 |
| 8. | 0 | 0 | 0 |
| 8. | 0 | 0 | 0 |
| 8. | 1 | 0 | 0 |
| 8. | 1 | 1 | 0 |
| 8. | 1 | 0 | 0 |
| 8. | 1 | 1 | 0 |
| 8. | 1 | 0 | 0 |
| 8. | 1 | 1 | 0 |
|  |  |  |  |
| Gy | TU212 non-transfection | 100nMN-CR | 100nM-TR |
| 0. | 1 | 1 | 1 |
| 0. | 1 | 1 | 1 |
| 0. | 1 | 1 | 1 |
| 0. | 1 | 1 | 1 |
| 0. | 1 | 1 | 1 |
| 0. | 1 | 1 | 1 |
| 0. | 1 | 1 | 1 |
| 0. | 1 | 1 | 1 |
| 0. | 0 | 0 | 0 |
| 0. | 1 | 0 | 0 |
| 0. | 1 | 0 | 0 |
| 0. | 1 | 1 | 0 |
| 0. | 1 | 1 | 0 |
| 0. | 1 | 1 | 0 |
| 0. | 1 | 0 | 0 |
| 2. | 1 | 1 | 1 |
| 2. | 1 | 1 | 1 |
| 2. | 1 | 1 | 1 |
| 2. | 1 | 1 | 1 |
| 2. | 1 | 1 | 1 |
| 2. | 1 | 1 | 1 |
| 2. | 1 | 1 | 0 |
| 2. | 0 | 0 | 0 |
| 2. | 0 | 0 | 0 |
| 2. | 0 | 0 | 0 |
| 2. | 1 | 1 | 0 |
| 2. | 1 | 0 | 0 |
| 2. | 1 | 0 | 0 |
| 2. | 1 | 1 | 0 |
| 2. | 1 | 0 | 0 |
| 2. | 1 | 1 | 0 |
| 4. | 1 | 1 | 1 |
| 4. | 1 | 1 | 1 |
| 4. | 1 | 1 | 1 |
| 4. | 1 | 1 | 0 |
| 4. | 1 | 0 | 0 |
| 4. | 0 | 0 | 0 |
| 4. | 0 | 0 | 0 |
| 4. | 0 | 0 | 0 |
| 4. | 0 | 0 | 0 |
| 4. | 0 | 0 |  |
| 4. | 1 | 1 | 0 |
| 4. | 1 | 1 | 0 |
| 4. | 1 | 0 | 0 |
| 4. | 1 | 1 | 0 |
| 4. | 1 | 0 | 0 |
| 4. | 1 | 1 | 0 |
| 6. | 1 | 1 | 1 |
| 6. | 1 | 1 | 1 |
| 6. | 1 | 0 | 0 |
| 6. | 1 | 0 | 0 |
| 6. | 0 | 0 | 0 |
| 6. | 0 | 0 | 0 |
| 6. | 0 | 0 | 0 |
| 6. | 0 | 0 | 0 |
| 6. | 0 | 0 | 0 |
| 6. | 0 | 0 | 0 |
| 6. | 1 | 0 | 0 |
| 6. | 1 | 1 | 0 |
| 6. | 1 | 0 | 0 |
| 6. | 1 | 1 | 0 |
| 6. | 1 | 1 | 0 |
| 6. | 1 | 1 | 0 |
| 8. | 1 | 1 | 1 |
| 8. | 1 | 0 | 0 |
| 8. | 1 | 0 | 0 |
| 8. | 0 | 0 | 0 |
| 8. | 0 | 0 | 0 |
| 8. | 0 | 0 | 0 |
| 8. | 0 | 0 | 0 |
| 8. | 0 | 0 | 0 |
| 8. | 0 | 0 | 0 |
| 8. | 0 | 0 | 0 |
| 8. | 1 | 0 | 0 |
| 8. | 1 | 1 | 0 |
| 8. | 1 | 0 | 0 |
| 8. | 1 | 1 | 0 |
| 8. | 1 | 0 | 0 |
| 8. | 1 | 1 | 0 |

**Comet Assay**


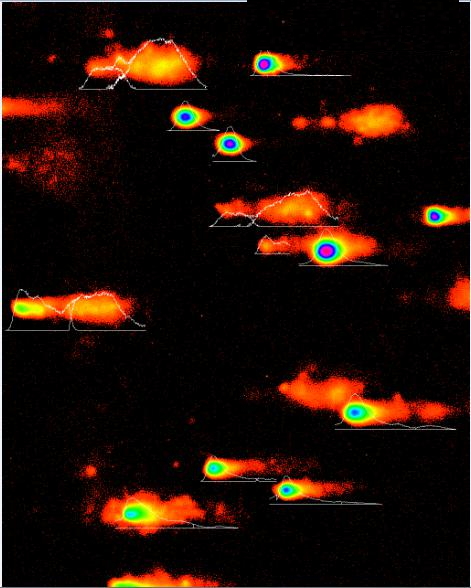

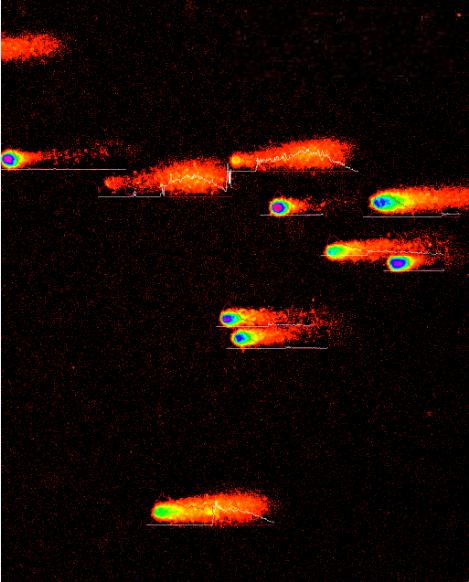

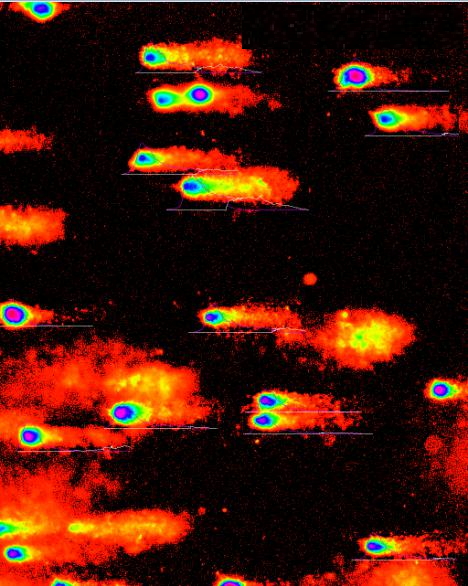


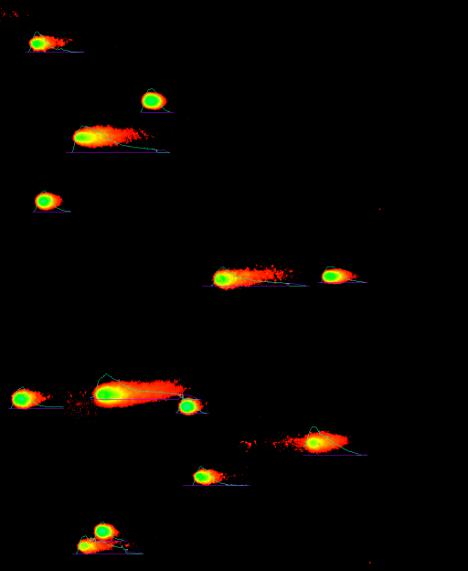

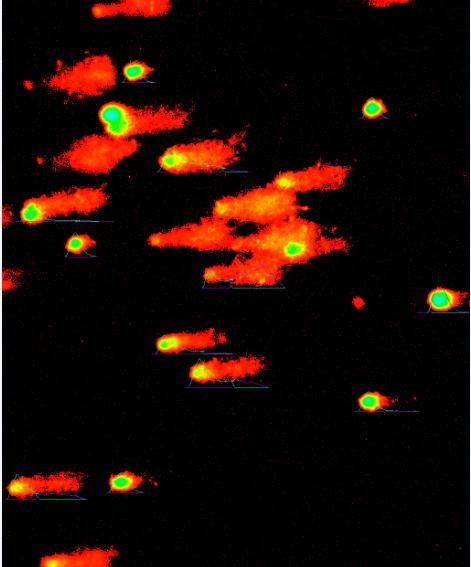

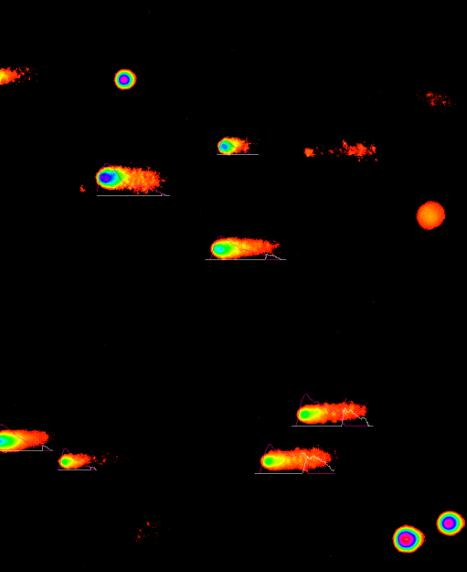


**Western Blotting**

|  | HEP-2 | | | | | | M2E | | | | | | TU212 | | | | | |
| --- | --- | --- | --- | --- | --- | --- | --- | --- | --- | --- | --- | --- | --- | --- | --- | --- | --- | --- |
| catenin | 58.2 | 56.42 | 54.43 | 123.07 | 129.6 | 127.74 | 71.61 | 75.22 | 76.17 | 183.2 | 175.28 | 178.35 | 85.02 | 77.31 | 85.5 | 144.44 | 139.76 | 143.12 |
| actin | 183.17 | 181.98 | 205.08 | 246.5 | 244.2 | 245.49 | 223.76 | 242.33 | 253.63 | 254.21 | 244.99 | 247.82 | 231.58 | 243.23 | 244.01 | 253.36 | 253.47 | 254.01 |
| **catenin** | **0.31774** | **0.31003** | **0.26541** | **0.49927** | **0.53071** | **0.52035** | **0.32003** | **0.3104** | **0.30032** | **0.72066** | **0.71546** | **0.71968** | **0.36713** | **0.31785** | **0.3504** | **0.5701** | **0.55139** | **0.56344** |
| yap1 | 98.37 | 98.49 | 96.27 | 157.24 | 163.61 | 159.8 | 142.07 | 159.45 | 171.01 | 240.15 | 236.51 | 244.61 | 178.62 | 175.17 | 172.93 | 241.36 | 251.69 | 247.88 |
| actin | 230.44 | 226.93 | 231.58 | 231.02 | 230.55 | 228.9 | 235.39 | 228.49 | 237.07 | 249.57 | 249.78 | 251.62 | 252.91 | 252.03 | 253.02 | 250.91 | 252.27 | 252.74 |
| **yap1** | **0.42688** | **0.43401** | **0.41571** | **0.68063** | **0.70965** | **0.69812** | **0.60355** | **0.69784** | **0.72135** | **0.96226** | **0.94687** | **0.97214** | **0.70626** | **0.69504** | **0.68346** | **0.96194** | **0.9977** | **0.98077** |
| jnk1 | 241.69 | 239 | 234.84 | 215.83 | 215.01 | 214.13 | 227.23 | 225.51 | 227.06 | 219.66 | 219.66 | 218.95 | 216.47 | 227.8 | 226.93 | 203.94 | 202.28 | 203.02 |
| p38 | 253.4 | 253.07 | 235.95 | 224.29 | 236.37 | 221.76 | 226.5 | 226.79 | 226.21 | 212.82 | 204.04 | 210.65 | 230.54 | 237 | 231.62 | 217.31 | 216.9 | 216.58 |
| actin | 254.85 | 254.07 | 254.89 | 252.51 | 250.83 | 252.6 | 251.65 | 253.21 | 253.39 | 254.08 | 254.58 | 254.58 | 247.2 | 250.43 | 251.35 | 250.43 | 252.32 | 253.04 |
| **jnk1** | **0.94836** | **0.94069** | **0.92134** | **0.85474** | **0.85719** | **0.8477** | **0.90296** | **0.8906** | **0.89609** | **0.86453** | **0.86283** | **0.86004** | **0.87569** | **0.90964** | **0.90284** | **0.81436** | **0.80168** | **0.80232** |
| **p38** | **0.99431** | **0.99606** | **0.92569** | **0.88824** | **0.94235** | **0.87791** | **0.90006** | **0.89566** | **0.89273** | **0.83761** | **0.80148** | **0.82744** | **0.93261** | **0.94637** | **0.9215** | **0.86775** | **0.85962** | **0.85591** |
